# Genotoxicity and Cytotoxicity of Gold Nanoparticles In Vitro: Role of Surface Functionalization and Particle Size

**DOI:** 10.3390/nano10020271

**Published:** 2020-02-06

**Authors:** Gerard Vales, Satu Suhonen, Kirsi M. Siivola, Kai M. Savolainen, Julia Catalán, Hannu Norppa

**Affiliations:** 1 Finnish Institute of Occupational Health, Työterveyslaitos, 00032 Helsinki, Finland; satu.suhonen@ttl.fi (S.S.); kai.m.savolainen@gmail.com (K.M.S.); julia.catalan@ttl.fi (J.C.); hannu.norppa@ttl.fi (H.N.); 2 Department of Anatomy Embryology and Genetics, University of Zaragoza, 50009 Zaragoza, Spain

**Keywords:** BEAS-2B, cellular uptake, DNA damage, functionalization, genotoxicity, gold nanoparticle, hyperspectral microscopy, in vitro, micronucleus, particle size

## Abstract

Several studies suggested that gold nanoparticles (NPs) could be genotoxic in vitro and in vivo. However, gold NPs have currently produced present a wide range of sizes and functionalization, which could affect their interactions with the environment or with biological structures and, thus, modify their toxic effects. In this study, we investigated the role of surface charge in determining the genotoxic potential of gold NPs, as measured by the induction of DNA damage (comet assay) and chromosomal damage (micronucleus assay) in human bronchial epithelial BEAS-2B cells. The cellular uptake of gold NPs was assessed by hyperspectral imaging. Two core sizes (~5 nm and ~20 nm) and three functionalizations representing negative (carboxylate), positive (ammonium), and neutral (poly(ethylene glycol); (PEG)ylated) surface charges were examined. Cationic ammonium gold NPs were clearly more cytotoxic than their anionic and neutral counterparts, but genotoxicity was not simply dependent on functionalization or size, since DNA damage was induced by 20-nm ammonium and PEGylated gold NPs, while micronucleus induction was increased by 5-nm ammonium and 20-nm PEGylated gold NPs. The 5-nm carboxylated gold NPs were not genotoxic, and evidence on the genotoxicity of the 20-nm carboxylated gold NPs was restricted to a positive result at the lowest dose in the micronucleus assay. When interpreting the results, it has to be taken into account that cytotoxicity limited the doses available for the ammonium-functionalized gold NPs and that gold NPs have earlier been described to interfere with the comet assay procedure, possibly resulting in a false positive result. In conclusion, our findings show that the cellular uptake and cytotoxicity of gold NPs are clearly enhanced by positive surface charge, but neither functionalization nor size can single-handedly account for the genotoxic effects of the gold NPs.

## 1. Introduction

Engineered nanomaterials (ENMs) have sparked enormous interest due to their unique properties associated with the nanoscale. The rise of nanotechnology is having a significant impact in a wide range of fields. New applications, improved efficiency, and new industrial processes are offering new tools and opportunities from medicine and diagnostics to battery technology and new materials [1].

However, the properties that make nanomaterials interesting are also those that raise concerns about toxicological potential during their interaction with biological systems. Due to their small size, ENMs can easily enter the cells and translocate across cells, tissues, and organs [2]. As size diminishes, the ratio of surface to total atoms or molecules increases exponentially. Due to their increased surface reactivity, nanomaterials are expected to have greater biological effects per given mass compared with larger particles [3].

Gold has historically been appreciated as currency and jewelry due to its lustrous, non-rusty, and scarce nature. It has mostly been considered an inert element in its bulk form [4]. Gold ENMs show promising applications mainly in the biomedicine field, due to their special properties in the nanoscale. Gold ENMs have a high X-ray absorption coefficient, with ease of synthetic manipulation (enabling precise control over the particle’s physicochemical properties), strong binding affinity to thiols, disulfides, and amines, and unique tunable optical and distinct electronic properties [5], which make them very interesting for biosensors and detection platforms, as well as for immunoassays, targeted drug delivery, bioimaging applications, and near-infrared photothermal ablation of microorganisms and cancer cells [6,7,8]. As many of the possible applications in biomedicine rely on a direct interaction between the gold ENMs and the target biological structure, determining the potential health effects of gold ENMs is also important.

Toxicological studies show that the potential toxic impact of gold nanoparticles (NPs) may be multisided and consequently hard to predict. Gold NPs are able to cause nano-specific toxic effects that differ from the effects of their bulk counterparts [9] and, in addition to particles size, surface chemistry and charged surface functional groups can also play a crucial role in determining toxic effects. Hence, thorough knowledge of the size-dependent toxic effects and surface properties of gold NPs is essential to predict the toxicity of gold NPs [10], with the objective of being able to correlate between particle parameters, experimental designs, and observed biological effects [11].

In the present study, we investigated the role of surface charge in determining the genotoxic potential of gold NPs in vitro, by analyzing their ability to induce DNA and chromosomal damage in human bronchial epithelial BEAS-2B cells. Two nominal core sizes (~5 nm and ~20 nm) and three functionalizations representing different surface charges—alkyl sodium carboxylate for negative charge, alkyl ammonium bromide for positive charge, and poly(ethylene glycol) (PEG) for neutral charge—were used in this study. Additionally, nanoparticle uptake was investigated by hyperspectral microscopy, to assess the effect of intracellular dose on the potential genotoxic and cytotoxic effects of gold NPs.

## 2. Materials and Methods

### 2.1. Gold Nanoparticles

Gold NPs were provided by the NANOSOLUTIONS consortium as ready-made suspensions in 0.5 M KCL in two sizes (~5-nm- and ~20-nm-core gold NPs), each functionalized with ligands that represented anionic (alkyl sodium carboxylate; –COOHNa), cationic (alkyl ammonium bromide; –N(CH_3_)_3_), or neutral charge (PEG-terminated; –PEG). The synthesis and functionalization procedures of the NPs, along with particle characterization (surface chemistry, transmission electron microscopy, dynamic light scattering, zeta potential, and ultraviolet–visible spectrophotometry), have previously been detailed [12]. Average primary particles sizes, as determined by TEM, were 2.5, 4.5 and 3.5 nm for the ~5-nm ammonium, carboxylate, and PEGylated gold NPs, respectively, and 15, 14, and 13 nm for the ~20-nm ammonium, carboxylate, and PEGylated gold NPs, respectively [12]. DLS in water showed average hydrodynamic diameter of 47, 77, and 32 nm for the ~5-nm ammonium, carboxylate, and PEGylated gold NPs, respectively, and 236, 30, and 20 nm for the ~20-nm ammo­nium, carboxylate, and PEGylated gold NPs, respectively [12]. In cell culture medium (with serum), the hydrodynamic diameter of the ammonium gold NPs and the 5-nm carboxylate NPs increased, presumably due to NP agglomeration in the presence of proteins [12]. 

### 2.2. Cell Culture

BEAS-2B cells (transformed human bronchial epithelial cells) were obtained from the American Type Culture Collection through LGC Promochem AB (Borås, Sweden). The BEAS-2B cells were grown in serum-free Bronchial Epithelial Growth Medium (BEGM; Clonetics, Walkerwille, MD, USA) at 37 °C in a humidified atmosphere of 5% CO_2_. In total, 60,000 cells were plated on 24-well plates (for cytotoxicity and comet assays; Nunc, Roskilde, Denmark) 24 h before treatment. For the micronucleus assay, 300,000 BEAS-2B cells were seeded 24 h before treatment in six-well plates (Nunc, Roskilde, Denmark).

### 2.3. Cytotoxicity

Cytotoxicity was determined by cell counts, using trypan blue (Sigma–Aldrich Chemie, Steinheim, Germany) to identify dead cells. This assay reflects all treatment-related effects (necrosis, cell-cycle delay, and apoptosis) that reduce the number of living cells. Semiconfluent BEAS-2B cell cultures on 24-well plates (culture area 1.9 cm^2^/well) were exposed to gold NP dispersions ranging from 1 to 250 µg/mL (treatment volume 500 μL per well; in BEGM) for 24 or 48 h. Three replicates per dose were performed. After the treatment, the wells were washed with phosphate-buffered saline (PBS; Corning Inc., Corning, NY, USA) and incubated with 250 µL of trypsin-EDTA (Gibco, Paisley, UK) 0.5% at +37 °C until cells were detached. Then, 0.5 mL of 10% fetal bovine serum (FBS; Gibco, Paisley, Scotland) in PBS was added to each well, and the contents were transferred to Eppendorf tubes which were centrifuged at 1100 rpm for 5 min at 4 °C. For each Eppendorf tube, the supernatant was discarded, and the cells were resuspended in 0.5 mL of 0.5% trypan blue in PBS and incubated on ice for 5 min. For the analysis, a sample was placed in a Bürker chamber, where four squares were counted, and the total number of cells in the well was calculated. Results were expressed as the percentage of cells compared to the negative control.

### 2.4. Genotoxicity

#### 2.4.1. Comet Assay

The comet (single-cell gel electrophoresis) assay was performed to study DNA strand breaks and alkaline labile sites as described by Lindberg et al. (2009) [13]. Briefly, semiconfluent BEAS-2B cells cultures on 24-well plates (culture area 1.9 cm^2^/well) were exposed to gold NP dispersions ranging from 1 to 250 µg/mL (treatment volume 500 μL per well; in BEGM) for 24 h, after which the cells were trypsinized and centrifuged at 1100 rpm for 5 min. For positive control, cells were treated with H_2_O_2_ (100 µg/mL; Sigma–Aldrich Chemie, Steinheim, Germany) for 15 min. In total, 10,000 to 30,000 cells were resuspended in 75 μL of molten (37 °C) 0.5% low-melting-point agarose (Bio-Rad Laboratories, Hercules, CA, USA). The resuspended cells in agarose were put onto dry microscope slides (Assistant, Sondheim/Röhn, Germany) precoated with 1% normal-melting agarose (International Biotechnologies, New Haven, CT, USA), and the agarose was allowed to solidify for 10 min. The slides were immersed in cold lysing solution (2.5 M NaCl, 100 mM EDTA, 10 mM Tris, 1% Triton X-100) for 1 h at 4 °C, after which they were transferred to an electrophoresis tank containing freshly made electrophoresis buffer (1 mM EDTA, 300 mM NaOH; pH > 13), where they were kept for 20 min at room temperature, to allow DNA unwinding. Electrophoresis was performed in the same buffer at room temperature for 15 min at 24 V and 300 mA (0.8 V/cm). The slides were then neutralized three times with 0.4 M Tris buffer (pH 7.5), air-dried, and fixed in methanol (Merck, Darmstadt, Germany). DNA was stained with ethidium bromide (2 μg/mL) in water for 5 min.

The slides were coded, and one scorer performed the comet analysis using a fluorescence microscope (Axioplan 2, Zeiss, Jena, Germany) and an interactive automated comet counter (Komet 5.5, Kinetic Imaging Ltd., Liverpool, UK). The percentage of DNA in the comet tail from 400 cells per experiment (two replicates per dose, two slides per replicate, 100 cells per slide) was used to measure the amount of DNA damage.

#### 2.4.2. Micronucleus Assay

The micronucleus assay was used to determine the potential of the gold NPs to induce chromosomal damage in BEAS-2B cells. The cell cultures were exposed to the gold NPs for 48 h in 2.5 mL of BEGM in six-well plates to keep the volume/well surface constant in all assays. Cytochalasin B (Cyt-B; 9 μg/mL; Sigma–Aldrich Chemie, Steinheim, Germany) was added 6 h after initiating the exposure, to induce binucleation of dividing cells. Mitomycin C (150 ng/mL; Sigma-Aldrich, Steinheim, Germany) was used as positive control.

After the exposure, the cells were rinsed with PBS twice and were incubated with 1 mL trypsin for 20 min, 10% FBS in PBS was added, and the cells were centrifuged at 1100 rpm for 6 min. The supernatant was removed, and the cells were washed with PBS. After centrifugation and removal of the supernatant, the cells were incubated in 5 mL of hypotonic solution, 50% Roswell Memorial Park Institute (RPMI)-1640 medium (Lonza, Basel, Switzerland) in distillated water, for 1 min. The cells were centrifuged and fixed in 3:1 methanol–acetic acid (Merck, Darmstadt, Germany) and, after another centrifugation and supernatant removal, they were fixed in 97% methanol–3% acetic acid. The cells were then spread on microscopic slides dropwise and were left to dry overnight. The slides were stained with acridine orange (32 μg/mL in Sørensen’s buffer, pH 6.8; Sigma–Aldrich Chemie, Steinheim, Germany) for 1 min and rinsed in Sørensen’s buffer thrice for 3 min. Finally, the slides were stained with 4′,6-diamidino-2-phenylindole (DAPI, 5 μg/mL; Sigma–Aldrich Chemie, Steinheim, Germany) for 5 min, then rinsed in tap water and allowed to dry. The stained and fixed slides were kept protected from light at 4 °C until analysis.

The slides were coded, and the frequency of micronucleated cells in 2000 binucleate cells per dose (1000 cells/replicate) was analyzed by one scorer using an Axioplan 2E Universal microscope (Zeiss, Jena, Germany). Binucleate cells were identified with a 40× lens using a green/red FITC/TRITC (fluorescein isothiocyanate/tetramethylrhodamine isothiocyanate) double filter.

### 2.5. Hyperspectral Imaging

The CytoViva hyperspectral imaging microscopy system (CytoViva, Inc., Auburn, AL, USA) mounted on an Olympus BX43 darkfield microscope (Olympus Corporation, Tokyo, Japan) was used to analyze the internalization of the different gold NPs by BEAS-2B cells. BEAS-2B cells were treated with 5 µg/mL NPs for 48 h (Cyt-B was added 6 h after the start of the treatment), cytospinned on microscope slides, and fixed in methanol. The slides were prepared for analysis by placing a drop of immersion oil and covering with a coverslip. Dark-field images were captured using a 100× objective, and images were processed by subsetting to bands 27–341 (wavelengths 430–840 nm), followed by a signal smoothing by the Savitzky–Golay filter (width 11 bands) and normalization for the lamp spectrum. To identify the spectra from the gold NPs in the samples, spectral libraries were created from each NP dispersion. A drop of the dispersion was placed on a slide and air-dried. A drop of immersion oil was added on the slide which was then covered with a coverslip and visualized at the same magnification as the BEAS-2B cells. The NP suspension sample slides were subjected to the same processing as the slides with the BEAS-2B cells. The spectral libraries were obtained by particle filtering, and negative control images were used to subtract possible overlapping spectra, to ensure that the spectral libraries only contained NP-specific spectra. Gold NP spectra were detected in the obtained cell images, by using the spectral libraries with the spectral angle mapper tool, to detect the unique spectral set of the NP of interest. In total, 15 cells were analyzed per gold NP type, by manually selecting the region of interest (ROI), to encompass all the cell area and counting the number of pixels per ROI matching with the library. The results were expressed as a percentage of matching pixels per cell.

### 2.6. Statistical Analysis

One-way ANOVA was applied to examine the percentage of DNA in tail in the Comet assay. Tukey’s test was used for a post hoc comparison of the means. Fisher’s exact test (two-tailed) was used to determine whether the treatment induced a statistically significant increase in the frequency of micronuclei as compared with the untreated control cultures. Dose–response in both assays was evaluated by linear regression analysis. Kruskal–Wallis test and post hoc Dunn’s test were used to compare the percentage of matching pixels per cell and among the different types of NMs, as well as in comparison with the untreated control cultures. All analyses were performed in GraphPad Prism 7.03.

## 3. Results

**Figure 1:**
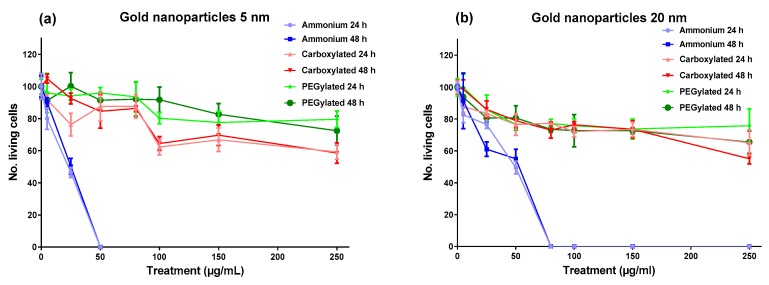
Number of living cells relative (%) to untreated control cultures after treatment of BEAS-2B cells with gold nanoparticles (core diameter: ~5 nm (**a**) or ~20 nm (**b**)) for 24 or 48 h. Dead cells were identified by trypan blue staining. The symbols represent means ± SD.

As shown in Figure 1, the cytotoxicity of gold NPs on BEAS-2B cells was driven by surface functionalization; the ammonium modification was clearly the most cytotoxic functionalization at both core sizes, with the 5-nm ammonium NPs reaching maximum cytotoxicity (no living cells) at 50 µg/mL and the 20-nm ammonium NPs reaching maximum cytotoxicity at 80 µg/mL. The carboxylated and PEGylated NPs showed low cytotoxicity for both core sizes, although the anionic 5-nm NPs were slightly more toxic than the neutral 5-nm NPs. The cytotoxicity of the gold NPs had very similar profiles after the 24-h and 48-h treatments, influenced mainly by functionalization.

**Figure 2:**
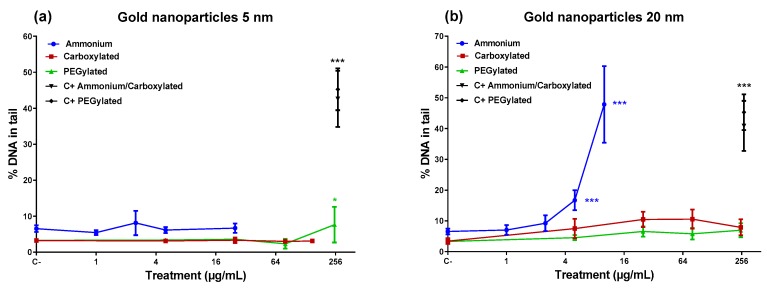
DNA damage expressed as tail intensity (% DNA in tail) in BEAS-2B cells after a 24-h treatment with gold nanoparticles (core diameter: ~5 nm (**a**) or ~20 nm (**b**)). Treatment is expressed as the antilog of Log_2_ of the dose. Here, 100 µM H_2_O_2_ was used as a positive control (C+; symbols on the right show results from the experiments carried out). The symbols represent means ± SD. Statistical significance in comparison with control cultures (one-way ANOVA): * *p* < 0.05; *** *p* < 0.001.

Figure 2 shows that the 5-nm PEGylated gold NPs produced a slight increase in DNA damage only at the highest dose tested, while neither the ammonium nor the carboxylate-functionalized 5-nm NPs caused DNA damage. However, the 20-nm ammonium NPs induced a high percentage of DNA damage at the top doses (5 and 10 µg/mL). The 20-nm carboxylated and the 20-nm PEGylated NPs did not affect the level of DNA damage. Only the 20-nm ammonium NPs showed a significant linear dose–response in inducing DNA damage (*R^2^* = 0.8316; *p* < 0.001).

**Figure 3:**
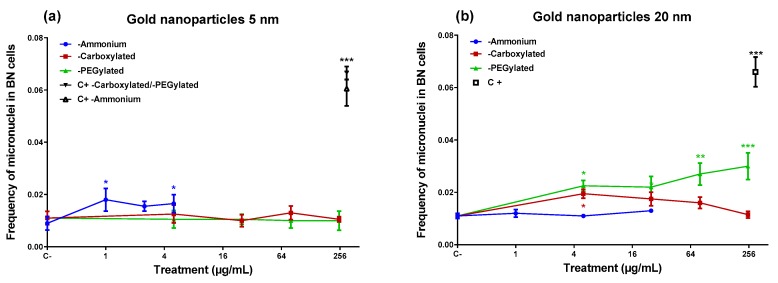
Micronucleus frequency in binucleated (BN) BEAS-2B cells after a 48-h treatment with gold nanoparticles (core diameter: ~5 nm (**a**) or ~20 nm (**b**)). Treatment is expressed as the antilog of Log_2_ of the dose. Mitomycin C (150 ng/mL) was used as a positive control (C+; symbol on the right). The symbols represent means ± SD. Statistical significance in comparison with control cultures (Fisher’s exact test, two-tailed): * *p* < 0.05; ** *p* < 0.01; *** *p* < 0.001.

In the micronucleus assay (Figure 3), the ammonium-functionalized 5-nm gold NPs were able to induce micronuclei (Figure 3), despite they did not produce DNA damage in the comet assay. On the other hand, the 20-nm ammonium-functionalized NPs (which induced DNA damage) did not generate an increase in micronuclei. The 20-nm PEGylated NPs induced micronuclei at three of the four doses tested, including the lowest and the highest dose, although they did not cause DNA damage in the comet assay. The carboxylated 20-nm NPs increased the frequency of micronuclei only at the lowest dose tested (5 µg/mL). None of the gold NPs that induced micronuclei showed a statistically significant linear dose–response (data not shown).

**Figure 4:**
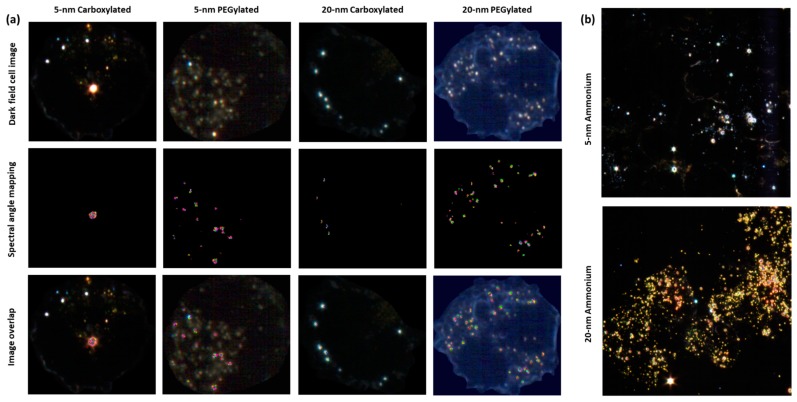
Hyperspectral image analysis of BEAS-2B cells after a 48-h treatment with 5 µg/mL gold nanoparticles (NPs). (**a**) Representative images of cells exposed to ~5-nm-core carboxylated, ~5-nm-core poly(ethylene glycol) (PEG)ylated, ~20-nm-core carboxylated, or ~20-nm-core PEGylated gold NPs. (**b**) Dark-field view of several cells exposed to ~5-nm- or ~20-nm-core ammonium-functionalized gold NPs.

**Figure 5:**
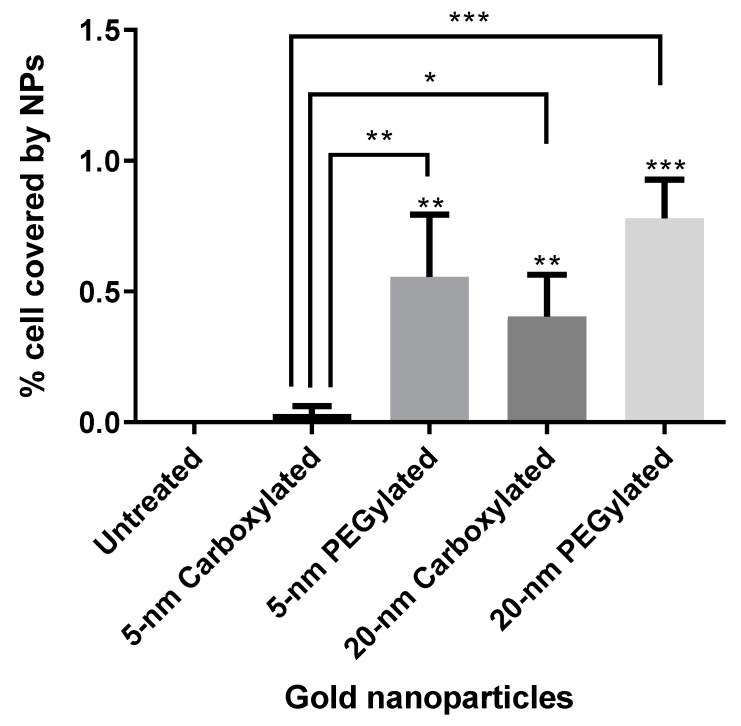
Percentage of BEAS-2B cells covered by gold nanoparticles (NPs) after a 48-h treatment with 5 µg/mL carboxylated and PEGylated gold NPs. In total, 15 cells were analyzed per treatment. The *p*-values are in comparison with untreated cultures or 5-nm carboxylated gold NPs (Kruskal–Wallis test with Dunn post hoc test): * *p* < 0.05; ** *p* < 0.01; *** *p* < 0.001.

Hyperspectral microscopy showed that all six types of gold NPs were taken up by BEAS-2B cells. NPs were mostly seen as clusters in the cytoplasm but not on the nucleus, which indicated that the NPs had been internalized. If the NPs had been attached to the cell membrane, a more uniform distribution of the particles, some covering the nucleus or locating on the outer edge of the cell membrane, would have been expected. The analysis of images obtained by hyperspectral microscopy revealed that cellular uptake was dependent on both size and functionalization. As shown in Figure 4a, the carboxylated and PEGylated gold NPs exhibited different patterns of distribution within the cells, with the PEGylated gold NPs being internalized in more numerous, smaller agglomerates than their carboxylated counterparts. In the case of the 20-nm carboxylated NPs, signal matching only recognized the agglomerates, which may have been partially due to a shift in the hyperspectral profile of the 20-nm carboxylated gold NPs inside the cells, as compared with the dispersion medium. In Figure 4b, it can be observed that the ammonium gold NPs were internalized in high numbers, preventing the visualization of individual cells. Figure 5 shows the results of the quantification of cell area covered by particles. The PEGylated NPs were internalized by the cells at higher amounts than the carboxylated NPs (statistically significantly only for the 5-nm size). The 20-nm carboxylated gold NPs were internalized more than their 5-nm counterparts, while no statistically significant difference was detected when comparing the 5- and 20-nm PEGylated gold NPs. The cell area covered by particles appeared to be much higher for the ammonium gold NPs than the other NPs, but this could not be quantified due to the difficulties in identifying individual cells.

## 4. Discussion

Although the cytotoxicity and genotoxicity of gold NPs have been examined in detail during the last few years, the features behind the potential genotoxic effect and biological interactions of gold NPs are still poorly understood. In this study, we investigated the role of particle functionalization, size, and internalization in determining the genotoxicity and cytotoxicity of gold NPs in a bronchial epithelial in vitro model. Our results suggest that functionalization is important in determining the cytotoxicity of gold NPs, with ammonium-functionalized gold NPs being cytotoxic at lower doses than carboxylated or PEGylated gold NPs, regardless of core particle size.

The genotoxicity of the gold NPs was not simply determined by a single functionalization. In the comet assay, the ammonium-functionalized 20-nm gold NPs induced DNA damage at 5 and 10 µg/mL while the other forms of the 20-nm NPs did not. However, for the 5-nm size, an increase in DNA damage was only obtained at the highest dose (250 µg/mL) of the PEGylated 5-nm gold NPs. A similar disparity was found in the micronucleus test where the 20-nm PEGylated gold NPs showed a clear micronucleus induction, while the 5-nm ammonium-functionalized form was able to induce micronuclei at two doses (1 and 5 µg/mL), and the 20-nm carboxylated NPs were positive at one dose (5 µg/mL). These findings suggest that both sizes of ammonium and PEGylated gold NPs have genotoxic potential, although none of these particle types were positive in both genotoxicity assays. The 5-nm carboxylated gold NPs were not genotoxic, and evidence on the genotoxicity of the 20-nm carboxylated gold NPs was restricted to a positive result at the lowest dose in the micronucleus assay. When interpreting the results, it has to be taken into account that cytotoxicity limited the doses available for the ammonium-functionalized gold NPs.

Surface functionalization is considered a key feature in the interaction, potential effects, and ultimate fate of ENMs within biological environments, as the surface-chemical properties of NPs play a crucial role in their interaction with cells [14]. NP surface properties are considered to determine the composition of biocorona around the particles, which in turn modulates the cellular uptake of particles and, therefore, the biological responses, consequently defining the potential safety and efficacy of ENMs [15]. Our finding of the high cytotoxicity of the ammonium-functionalized gold NPs is in line with a recent study on the same set of gold NPs, where the ammonium gold NPs were cytotoxic to human monocytic leukemia THP-1 cells at 25 µg/mL, while the carboxylated and PEGylated forms were not cytotoxic within the dose range tested [12]. An omics analysis suggested that the ammonium NPs were able to significantly inhibit mitochondrial respiration. Goodman et al. [16] found that 2-nm-core cationic gold NPs caused cytotoxic effects in all cell lines investigated, while anionic gold NPs were not cytotoxic at the doses included. However, some studies suggested that negatively charged gold NPs show similarly high toxicity to positively charged gold NPs. In human keratinocyte HaCaT cells, triphenyl phosphine-stabilized 1.5-nm gold NPs with a positively charged (trimethylammoniumethanethiol) or negatively charged (mercaptoethanesulfonate) ligand evoked a greater toxic response than neutral (mercaptoethoxyethoxyethanol ligand) gold NPs of the same size [17]. Paino et al. [18] investigated the genotoxicity (comet assay) and cytotoxicity of cationic (polyamidoamine dendrimer-capped) and anionic (sodium citrate-capped) gold NPs (diameter ~7 nm) in HepG2 cells and human peripheral blood mononuclear cells (PBMCs), and they found that both particle types were genotoxic and cytotoxic at very low doses. PBMCs were less sensitive to the DNA-damaging and cytotoxic effects of the gold NPs than HepG2 cells. In human lung adenocarcinoma A549 cells, positively and negatively charged and neutral gold NPs (diameter 2–3 nm) all induced DNA damage after a 24-h treatment (comet assay), although the strongest genotoxic effect was observed for the positively charged gold NPs [19].

In the present study, the strong cytotoxicity of the cationic ammonium gold NPs was associated with an extremely high cellular uptake. The amount of internalization was lower for the anionic carboxylated NPs than for the neutral PEGylated NPs of both sizes, with the 5-nm carboxylated NPs showing a clearly lower cellular uptake than all other particle types. Nevertheless, cytotoxicity profile was similar for the carboxylated and PEGylated NPs. The distribution of the internalized particles was visually different for the PEGylated and carboxylated NPs, with the former showing more small agglomerates than the latter. Interestingly, in a study about the internalization of gold NPs in the same cell line (BEAS-2B; albeit with 10% serum in the culture media), the authors observed that the mechanism of endocytosis pathway dictated intracellular localization and subsequent toxicity [20]. It was earlier observed that the cytotoxicity of gold particles (length ~165 nm) with poly(acryloyl-l(d)-valine coating of different surface chirality depended on their cellular uptake and production of intracellular reactive oxygen species [21].

In our study, the two core sizes (~5 nm and ~20 nm) of the gold NPs did not markedly differ from each other as concerns cytotoxicity. Size did not either play a consistent role in determining genotoxicity. The carboxylated 5-nm gold NPs were not genotoxic, but the other particle types showed at least some activity in either of the assays. The size of gold ENMs was earlier linked to differential toxic effects in vitro. Au55 gold clusters (diameter 1.4 nm) were cytotoxic at low doses through an intense interaction with DNA [9], but 15-nm gold NPs required 60–100 times higher doses for cytotoxicity in the same experimental setting [22]. In HepG2 human hepatocellular carcinoma cells, 5-nm NPs induced a dose-dependent increment in DNA damage after a 24-h exposure using the comet assay, whereas 20-nm and 50-nm gold NPs did not increase DNA damage at the doses tested [23]. In another study with HepG2 cells, 10-nm, 30-nm, and 60-nm gold NPs induced DNA damage, with the smallest NPs showing the highest effect [24]. When immortalized normal human bronchial epithelial HBEC3-kt cells were exposed to 1, 10, and 20 μg/mL (0.3–6.3 μg/cm^2^) gold NPs (5 and 50 nm) in milli-Q water for 48 h, only the 5-nm (not the 50-nm) particles induced DNA damage (comet assay), while neither of the particle types induced micronuclei [25].

In cultures of peripheral blood lymphocytes (PBLs) and murine Raw264.7 macrophages treated with citrate-capped gold NPs, 5-nm NPs were more cytotoxic than 15-nm NPs, but size did not play a pivotal role in determining cytotoxicity when the dose of gold NPs was expressed as the number of NPs [26]. However, for genotoxicity, size was a fundamental factor for both dose metrics and cell types, with the 15-nm gold NPs showing a more severe micronucleus induction than the smaller particles [26]. Enea et al. [27] studied the cytotoxicity of gold NPs differing in size (14 nm and 50 nm), shape (spheres and stars), and coating (11-mercaptoundecanoic acid (MUA) and sodium citrate; both negatively charged) in human cerebral microvascular endothelial cells (hCMEC/D3), and they observed that the smaller gold NPs were more cytotoxic than the larger gold NPs when compared at the same gold dose. However, when dose was expressed as the number of particles/mL, a higher degree of cytotoxicity was noted for the larger NPs. Moreover, cytotoxicity was greater for star- than sphere-shaped gold NPs, and the citrate coating resulted in higher cytotoxicity than the MUA coating. Another study investigating the same functionalizations (citrate and MUA) of 20-nm gold NPs in HepG2 cells found an increase in DNA damage (comet assay) after exposure to the citrate-coated NPs at doses that were not genotoxic for the MUA-coated NPs [28].

Size was also observed to play a key role in the toxicity and biodistribution of gold ENMs in vivo. When Sprague–Dawley rats were exposed by inhalation to 13-nm or 105-nm gold NPs for five days (6 h/day), both types of NPs deposited mainly in the lungs, but translocation from the lungs to secondary target organs was significantly higher with the smaller than the larger gold NPs [29]. In rats, intratracheal instillation of gold NPs differing in primary particle size (2, 20, and 200 nm; single instillation of 18 μg in 0.5 mL) did not result in genotoxic effects in the lungs (comet assay) or in the bone marrow (micronucleus assay) 72 h after the treatment, although it was not confirmed if the test materials reached the bone marrow [30]. Other systemic or local adverse effects were also not observed at the dose applied. PEG-coated gold NPs (5–60 nm) administered to male mice via intraperitoneal injection showed size-dependent accumulation, with 5-nm and 10-nm particles mainly accumulating in the liver and 30-nm particles mainly accumulating in the spleen, while the 60-nm particles had a limited accumulation in various organs [31]; however, the 10-nm and 60-nm NPs had a higher influence than the 5-nm and 30-nm NPs on standard hematology markers. Talamini et al. [32] studied the biodistribution of carboxy-terminated gold NPs (10-nm and 50-nm spherical and 50-nm rod- and star-shaped NPs), and they found that not only size but also shape greatly influenced the accumulation kinetics and excretion of gold NPs in filter organs, with spherical and star-like gold NPs showing the same percentage of accumulation but a different localization in the liver, whereas only the star-like gold NPs were able to accumulate in the lungs. In *Daphnia magna*, positively charged gold ENMs were orders of magnitude more toxic than negatively charged gold ENMs [33]. Gold NPs representing different sizes and surface charges, intravenously injected into rats, showed differential accumulation in various organs and tissues, depending on the features of the NPs, mediated by dynamic protein binding and exchange [34]. When rats were exposed to gold NPs by intra-esophageal instillation, the highest accumulation in secondary organs was mostly found for 1.4-nm NPs, and negatively charged NPs showed a higher accumulation than positively charged NPs. However, 18-nm gold NPs accumulated in the brain and heart in higher amounts than NPs of other sizes [35].

The reactivity of NPs has the potential to interfere with several in vitro assays [36]. For example, 14-nm citrate-stabilized gold NPs (negative charge) were observed to have the ability to interfere with the alkaline comet assay during critical steps where cell membranes are lysed, and the intracellular NPs have the potential to directly interact with the DNA [37]. This might produce false positive results. Most data on the induction of primary DNA damage by gold NPs are based on the alkaline comet assay. For instance, in A549 cells, 3-nm positively charged gold NPs induced a very strong response in the alkaline comet assay, but only a modest effect in fluorometric detection of alkaline DNA unwinding (FADU) and in the neutral comet assay (depicting DNA double-strand breaks), and no effect in the immunofluorescence analysis of γH2AX foci, another technique for DNA double-strand breaks [19]. These findings were considered to suggest that gold NPs predominantly induce alkali-labile sites which are detected in the alkaline comet assay but not in the other three assays [19]. Interference of gold NPs with the comet assay procedure might be an explanation for the high effect we observed at low doses of the 20-nm cationic gold NPs. As shown by dark-field microscopy, the cells were filled with gold NP agglomerates, due to an extremely high internalization of the NPs, rendering it possible for them to interact with DNA during the comet assay procedure. Furthermore, the elevated DNA damage induction by the 20-nm cationic NPs was not accompanied by an increase in micronuclei. However, the opposite was found for the 5-nm cationic NPs which induced micronuclei but not DNA damage, despite the internalization of these NPs was also very high. Another remarkable fact was that the 20-nm PEGylated gold NPs induced micronuclei, whereas the 5-nm PEGylated NPs did not. Micronucleus formation cannot be explained by the same technical problem that may affect the comet assay. An increase in micronuclei is, therefore, an indication of a true genotoxic effect. Micronuclei are formed from chromosomal fragments (clastogenic effect resulting from DNA-damage) or lagging whole chromosomes (aneugenic effect). In human PBLs and mouse RAW264.7 macrophages, micronuclei induced by 5-nm and 15-nm gold NPs were more often centromere-positive than -negative, suggesting a stronger aneugenic than clastogenic effect [18]. A clastogenic influence was further indicated by the fact that the gold NPs increased the frequencies of nucleoplasmic bridges (reflecting structural chromosomal rearrangements) in PBLs (5-nm NPs) or in both cell types (15-nm NPs) [18]. Thus, at least some gold NPs appear to show DNA-damaging capacity in assays that are not affected by the possible comet assay artefact. In the present study, the 5-nm ammonium and 20-nm PEGylated gold NPs were able to increase the frequency of micronuclei and should, therefore, be considered genotoxic in our in vitro system. The positive result obtained with the 20-nm ammonium and 5-nm PEGylated gold NPs in the comet assay may have been affected by NP interference with the assay protocol, and it should be viewed with caution.

## 5. Conclusions

Our findings show that the cytotoxicity of gold NPs in BEAS-2B cells is greatly enhanced by the ammonium functionalization, reflecting the very high cellular uptake of the ammonium gold NPs. Neither functionalization nor size is able to single-handedly account for the genotoxic effects of the gold NPs in BEAS-2B cells. The 5-nm ammonium and 20-nm PEGylated gold NPs increase the frequency of micronuclei and are, therefore, genotoxic. The 20-nm ammonium and 5-nm PEGylated gold NPs induce DNA damage, but it is unclear whether this effect is real or due to gold NP interference with the comet assay. The 20-nm carboxylated gold NPs show an equivocal effect, with a slight micronucleus induction only at the lowest dose, while the 5-nm carboxylated gold NPs are not genotoxic, and they also have the lowest cellular uptake.
